# Psychological determinants of successful practical teaching: personality traits, self-efficacy, and subjective perception in a hands-on clinical skills course

**DOI:** 10.1186/s12909-026-09788-2

**Published:** 2026-07-02

**Authors:** Andrea Lenes, Martin Klasen, Gabriel Bohorquez-Mendoza, Judith Gecht, Saša Sopka, Lina Vogt

**Affiliations:** 1 AIXTRA – Competence Center for Training and Patient Safety, Medical Faculty, RWTH Aachen, Forckenbeckstraße 71, Aachen, 52074 Germany; 2 Department of Anesthesiology, Medical Faculty, University Hospital RWTH Aachen, RWTH Aachen, Aachen, Germany

**Keywords:** Clinical skills, Teaching, Personality traits, Self-efficacy, Academic performance, Physical examination, Psychological safety, Structured feedback

## Abstract

**Purpose:**

Physical examination is central to undergraduate medical education, yet students may differ in how they experience group-based clinical skills training. This study investigated associations between personality traits, self-efficacy, comfort in group learning settings, and self-assessed learning success in a peer-led physical examination course with standardized patients.

**Methods:**

Medical students completed an online survey before and after the course. Personality traits were assessed using the NEO Five Factor Inventory, and self-efficacy was measured with the General Self-Efficacy Scale. Custom-designed scales assessed comfort in group learning settings and self-assessed confidence in eight physical examination skills. Self-assessed learning success was defined as the individual change in confidence between the two measurement points.

**Results:**

Comfort in group learning settings was positively associated with self-efficacy and negatively associated with neuroticism. After Bonferroni correction, no significant associations were found between comfort and extraversion, conscientiousness, agreeableness, or openness. neuroticism and self-efficacy were substantially negatively correlated. Comfort in group learning settings was positively associated with self-assessed learning success. Exploratory cluster analysis suggested two student profiles differing in neuroticism/self-efficacy, comfort, and self-assessed learning success.

**Conclusion:**

The findings indicate that students’ experiences in group-based physical examination training are associated with individual psychological characteristics, particularly neuroticism and self-efficacy. Clinical skills training should foster psychological safety, structured feedback, scaffolded practice, and low-stakes learning opportunities to support students with different learner profiles. Future studies should include objective performance measures to complement self-assessed outcomes.

**Supplementary Information:**

The online version contains supplementary material available at 10.1186/s12909-026-09788-2.

## Introduction

Physical examination is a core competency in clinical practice and is central to undergraduate medical education [1–3]. Nevertheless, studies continue to report concerning performance gaps among final-year medical students and recent graduates in this domain [4, 5]. These findings highlight a persistent challenge: how to ensure that students achieve sustainable competence in physical examination before entering clinical practice.

Teaching physical examination is complex, requiring students to integrate theoretical knowledge with manual and interpersonal skills. Numerous instructional methods—such as bedside teaching, simulation with or without standardized patients (SPs), and peer-led formats—have been introduced to support this learning [6, 7]. Nevertheless, the question remains whether these approaches are equally suited for all students.

As previous research has explored, certain personality factors have a strong predictive effect on academic achievement and performance among medical students [8–10]. For instance, the fundamental personality factors of the Big Five model [11] - neuroticism, extraversion, openness, agreeableness, and conscientiousness - have been shown to relate to individual learning preferences and educational outcomes, with Kolb’s learning styles being among the most frequently investigated frameworks in this context [12, 13]. Moreover, extraversion and conscientiousness have been found to positively correlate with academic success, whereas emotional stability, as the counterpart to neuroticism, has shown no or only minimal influence in this regard [14, 15]. It is noteworthy that these personality traits are not directly related to general intelligence [16], suggesting that they interact with learning through motivational, behavioral, and self-regulatory mechanisms rather than through cognitive ability alone.

One such mechanism is self-efficacy, defined as an individual’s belief in their capability to successfully perform a specific task. Introduced by Bandura, self-efficacy is not merely a general confidence trait, but a context- and task-specific construct that influences motivation, persistence, emotional responses, and the willingness to engage with challenging learning situations [17–19]. Importantly, self-efficacy is related to the Big Five factors [20]; specifically, lower neuroticism and higher extraversion, openness, agreeableness, and conscientiousness are associated with higher self-efficacy. In educational contexts, self-efficacy has repeatedly been identified as an important predictor of academic achievement and skill acquisition [21].

Personality traits and self-efficacy may be particularly relevant in medical education, where students are required not only to acquire theoretical knowledge but also to perform practical and interpersonal skills under observation. Previous studies have shown that self-efficacy plays an important role in medical training, including students’ confidence in clinical reasoning, communication, procedural skills, and patient encounters [22]. In the context of physical examination, self-efficacy is especially relevant because students must translate theoretical knowledge into hands-on performance while interacting with patients or standardized patients. Low self-efficacy in this domain may therefore contribute to avoidance, discomfort, or reduced perceived learning success, whereas high self-efficacy may facilitate active participation, repeated practice, and more positive learning experiences.

These individual differences may be particularly important in collaborative, practice-based settings, where students must perform socially and under observation - a common situation in many medical education courses. Based on previous findings, it seems plausible that students with certain personality profiles and higher task-specific self-efficacy may subjectively benefit more from this type of teaching setting than others. Conversely, students with lower self-efficacy or personality traits associated with social reservation or performance-related discomfort may experience such settings as less supportive. This course - a peer-led physical examination program with standardized patients embedded in the medical curriculum at RWTH Aachen University—offers a structured group learning environment designed to bridge theory and practice. It is a hands-on course in a group setting, designed to equip future practitioners with crucial structured physical examination skills. Thus, the initiative represents an educational approach specifically created to address the gap between theoretical knowledge and its practical application in clinical contexts.

Over the last years, formative assessment and practice-based learning formats have become widespread approaches in medical education [23]. Such formats integrate elements of problem-based learning [24, 25] and team-based learning [26–28], both of which emphasize active participation, collaboration, and feedback. Therefore, the course can be considered a representative example of practical learning in a group setting. The formative, hands-on nature of these learning environments raises a key pedagogical question: how do personality traits and self-efficacy shape students’ comfort and perceived success in such settings? As personality traits influence behavior in social situations, they may also affect how students engage with and experience social learning contexts. This may be particularly true when such situations are combined with the demonstration of potentially self-relevant skills, as is the case in physical examination training. For example, individuals with lower extraversion, a trait characterized by more reserved behavior in social situations, may feel less comfortable in group-based, observed practice settings. Similarly, students with lower self-efficacy regarding physical examination may perceive these settings as more challenging, independent of their actual ability. Therefore, our study explored how personality traits and self-efficacy are related to comfort in group-learning settings and self-assessed learning success in practical group-based learning situations. By examining these associations, we aimed to provide a stronger basis for developing personalized and learner-sensitive teaching strategies in undergraduate medical education.

## Methods

Participants were students in their 3rd study semester of medicine at the Medical Faculty of RWTH Aachen University participating in a physical examination course. At the time of the study, age and gender of the students was not assessed; however, these data were recorded at the beginning of their first semester in October 2021, i.e. 14 months before data acquisition for the present study. At this time point, median age of the entire student cohort was 19 years (IQR = 4 years). Concerning gender, 64.5% were female, 35.1% male, and 0.4% other. The course prepares the students for more advanced examination courses and comprises six one-hour sessions, covering the fundamental physical examination skills. The first five sessions follow an organ-based approach. In the first session, the students study how to perform the overall patient inspection and the examination of the back and extremities. Subsequent sessions are devoted to the examination of the head and neck, followed by focused assessments of the heart and lungs, and the abdomen. The fifth session covers the examination of the range of motion, strength, pulses, and reflexes. The curriculum culminates in a sixth session where students perform a head-to-toe examination on SPs, synthesizing skills acquired in earlier sessions. During the course, students receive individual feedback from peer teachers and, in the last session, also from SP and fellow students. (see Appendix 1)

Before the course begins, the students receive a script booklet that supports their learning process, containing all necessary materials for preparation and revision. The course places a strong emphasis on hands-on skills and peer-to-peer learning, where students practice examination techniques in small groups on SP under the guidance of fellow student tutors. Besides enhancing knowledge acquisition, this approach also fosters interpersonal abilities, which are essential for medical practice. The collaborative learning atmosphere specifically nurtures both the technical skills, and the empathetic approach required in clinical settings.

To evaluate the effectiveness of the course and its impact on student learning, we utilized a comprehensive methodological framework. Data was collected through a customized online evaluation survey administered before and after the course from December 6, 2022, to February 2, 2023. The questionnaire comprised the following elements:

- 30-item short version of the NEO Five Factor Inventory (NEO-FFI-30) [29]. The well-established NEO-FFI inventory assesses the personality traits openness, conscientiousness, extraversion, agreeableness, and neuroticism [30]. Briefly, the underlying personality model was empirically derived from personality-related self-descriptions using factor analysis; the five basic personality factors (“Big Five”) are largely independent from each other and have been replicated in various cultures and contexts. For the present study, we used a validated 30-item short version in German, which maintains the good psychometrical properties (internal consistencies (Cronbach’s Alpha) between 0.67 and 0.81) and construct validity of the original 60-item scales (Pearson correlations of 30-item and 60-item scales between 0.88 and 0.91) while being more economic. The objective was to investigate how these traits influence students’ learning capabilities and adaptability within the course.
- The General Self-Efficacy Scale (SWE) [31]: This 10-item inventory was used to gauge the students’ belief in their ability to excel in situations, especially in unfamiliar and challenging clinical settings. The one-factor scale has a good reliability (Cronbach’s Alpha between 0.8 and 0.9 for various samples) and convergent validity has been confirmed by correlations with related constructs [32].
- A custom designed scale, encompassing 4 items, assessed students’ general comfort levels during learning of clinical skills in a group setting. This approach was chosen since no validated scale for this purpose matching our needs could be identified. This scale has been constructed by the study authors according to face validity of the items; psychometric properties have not been assessed a priori. The author team was multiprofessional, including experts from the fields of medical anamnesis, medical teaching, psychology, and psychometrics to assure the best possible outcome. Item development took place in a group decision process. First, relevant content aspects were identified; second, these were transformed into clearly understandable items. The aim was to develop items describing typical situations in group-based learning situations with a possible influence on the student’s comfort. The final items described four typical situations, all of which were given in the physical examination course (practical exercise in front of a group; examining a fellow student; examining a simulated person; receiving feedback in front of a group).
- Self-assessed learning success for eight different examination skills. The participants were in their 3rd study semester and had been in touch with all these examination skills in their studies before the course started. Based on these skills, an 8-item scale was constructed, measuring the subjective confidence for performing the respective skill on a 6-step Likert scale. This subjective confidence was assessed before and after the course. Subsequently, self-assessed learning success of each participant was defined as the individual difference in total scores between the two time points, i.e. as the gain in subjective confidence across all eight examination skills. Like the previous scale, no existing inventory for this purpose could be found; it was constructed according to face validity and not validated before.

Data analysis involved correlation assessments and t-tests to determine relationships between personality traits on the one hand and subjective perception of academic outcomes on the other hand. This analysis aimed to identify predictors of academic success and areas for curriculum improvement. The threshold for statistical significance was determined as α = 0.05 for each individual test. For research question 1, Bonferroni’s correction method for multiple comparisons was applied to adjust the significance level to avoid false positive results, resulting in an adjusted threshold of α = 0.008. Individual sample sizes varied slightly for each correlation due to missing data points (please see results below).

Before participating in the online evaluation, all students were informed about the background and the purpose of the evaluation, the voluntariness of participation, as well as on the details of data acquisition in written form. Written informed consent was obtained from all participants prior to study participation.

Analysis of the data was guided by the following hypotheses:

- H1: General comfort in group learning settings is correlated with general self-efficacy. Specifically, previous findings suggest that this correlation is positive in nature.
- H2: General comfort in group learning settings is correlated with one or more of the Big 5 personality traits. Although previous findings suggest such relationships, we refrained from hypotheses for specific personality traits.
- H3: General comfort in group learning settings is positively correlated with self-assessed learning success. This hypothesis was derived from H1; specifically, we assumed that self-efficacy, positive emotions in learning situations, and the perception of successful learning go together.

## Results

H1: General comfort in group learning settings is correlated with general self-efficacy. As hypothesized, general comfort in group learning settings was positively associated with self-efficacy (*r* = .309; *p* < .001; *n* = 144).

H2: General comfort in group learning settings is correlated with one or more of the Big 5 personality traits. After Bonferroni correction (see above), the only trait variable showing significant correlations with general comfort in group learning settings was neuroticism (*r* = − .315; *p* < .001; *n* = 146). No significant correlations were observed with extraversion (*r* = .203; *p* = .016; *n* = 140), conscientiousness (*r* = .184; *p* = .026; *n* = 145), agreeableness (*r* = − .051; *p* = .539; *n* = 145) or openness (*r* = − .059; *p* = .489; *n* = 141).

For explorative purposes, we calculated furthermore the correlation between neuroticism and self-efficacy. We found a substantial negative correlation (*r* = − .518; *p* < .001; *n* = 146), suggesting that high neuroticism frequently goes along with low self-efficacy. Indeed, combining these two in a composite variable yielded the highest (negative) correlation with general comfort in group learning settings (*r* = − .382; *p* < .001; *n* = 146).

The correlations with general comfort in group learning settings are shown in Table 1.

**Table 1:** Correlations with General Comfort in Group Learning Settings. The combination of neuroticism and self-efficacy showed the highest correlation with general comfort in group learning settings

Correlations with General Comfort in Group Learning Settings								
		Neuroti-cism	Extra-version	Open-ness	Agreeable-ness	Conscien-tiousness	Self-Efficacy	Neuroticism + Self-Efficacy
General Comfort in Group Learning Settings	r	− 0.315^***^	0.203^*^	-0.059	-0.051	0.184^*^	0.369^***^	− 0.382^***^
	p	< 0.001	0.016	0.489	0.539	0.026	< 0.001	< 0.001
	N	146	140	141	145	145	193	144

* *p* < .05; ** *p* < .01; *** *p* < .001

H3: General comfort in group learning settings is positively correlated with self-assessed learning success.

These two variables showed a substantial positive correlation (*r* = .355; *p* < .001; *n* = 142), indicating that feeling comfortable in group learning situations goes along with higher self-assessed learning success.

**Fig. 1:**
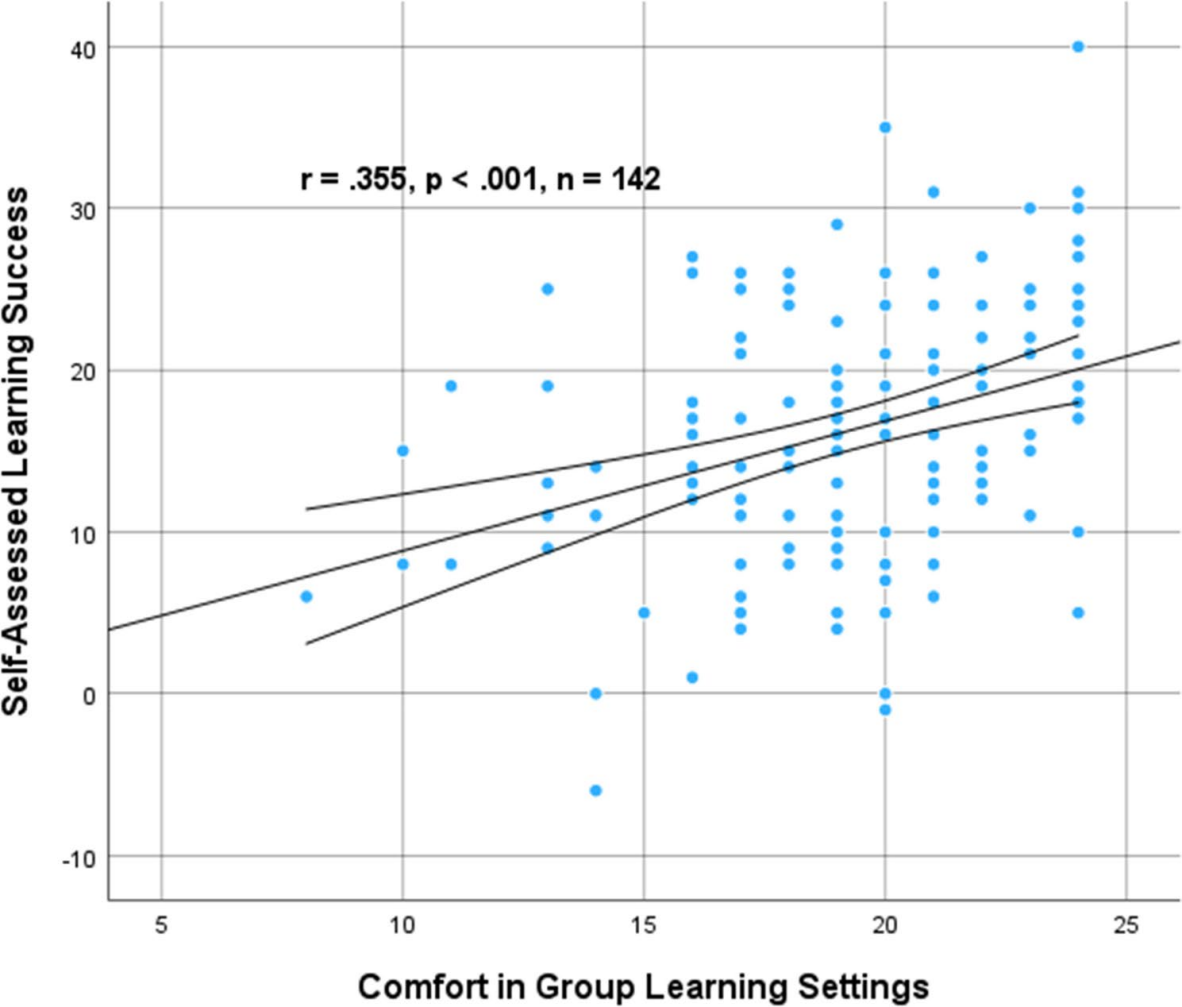
Positive correlation of general comfort in group learning settings with self-assessed learning success, including regression line and 95% CI. Higher values on x and y axes indicate higher comfort and higher learning success, respectively

The correlation pattern of general comfort in group learning settings and self-assessed learning success is depicted in Fig. 1.

### Explorative analysis: k-means cluster analysis

**Fig. 2:**
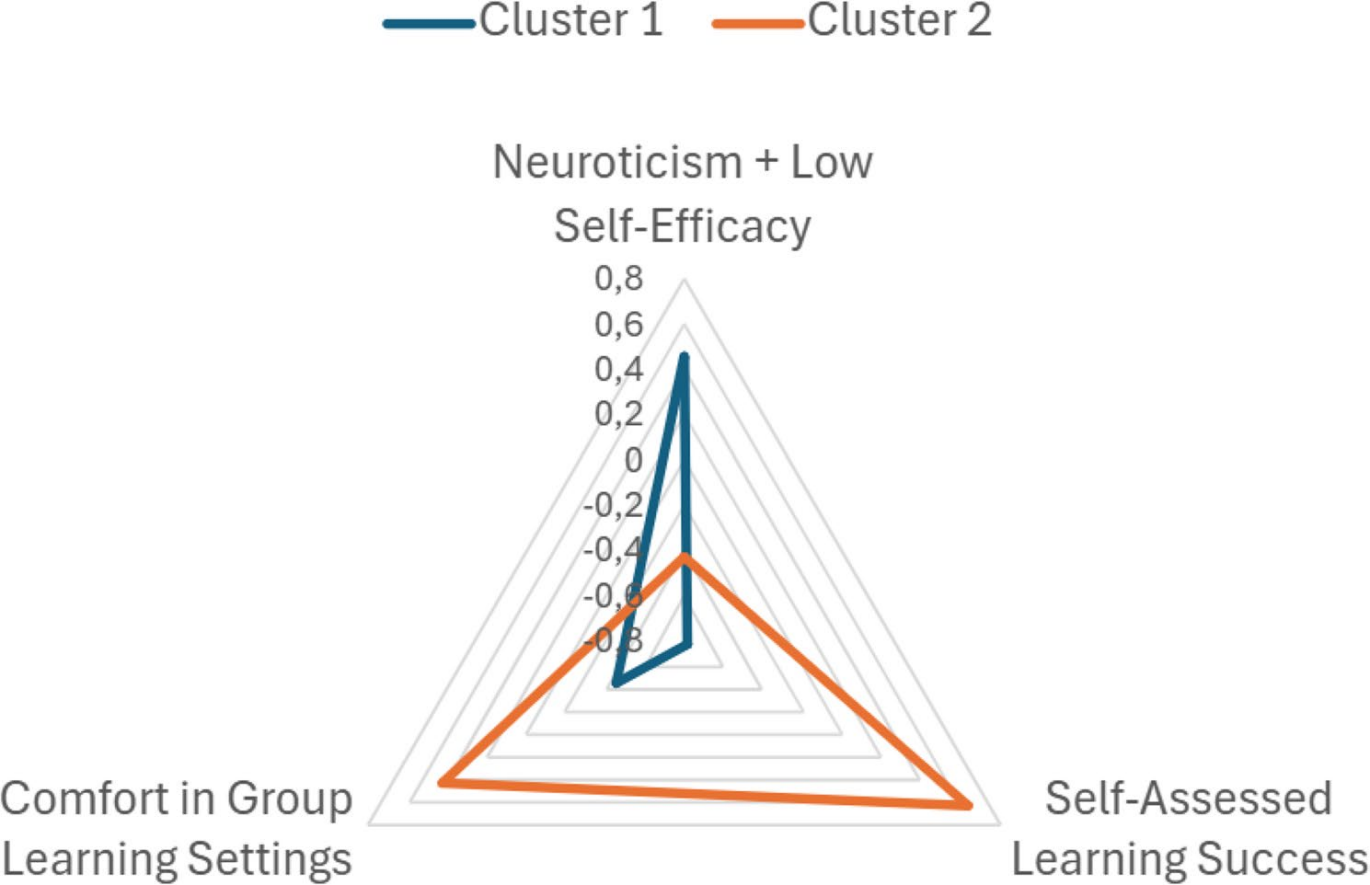
Cluster profiles. The results of the k-means cluster analysis suggest two different clusters of persons, each with a distinct variable profile. Mean variable values for the two clusters are shown in blue and orange (all variables z standardized for visualization purposes)

Given the correlational pattern of neuroticism, self-efficacy, comfort in group-learning settings, and self-assessed learning success, we concluded that these variables might define two distinct groups of persons. We therefore performed an explorative k-means cluster analysis for k = 2 clusters with three variables (composite neuroticism + self-efficacy; self-assessed learning success; comfort in group learning settings). All three variables were significant.

(all *p* < .001), indicating that the variables characterized two different clusters of persons. Cluster 1 was characterized by high neuroticism combined with low self-efficacy, low comfort in group learning settings, and low self-assessed learning success. Cluster 2, in turn, was characterized by low neuroticism combined with high self-efficacy, high comfort in group learning settings, and high self-assessed learning success. The cluster profiles are depicted in Fig. 2.

## Discussion

This study investigated the relationship between personality traits, self-efficacy, and comfort in group learning settings, as well as the association between comfort in these settings and self-assessed learning outcomes. The results highlight how individual psychological characteristics - particularly neuroticism and self-efficacy—shape medical students’ experiences in group-based clinical skills training. Specifically, higher neuroticism was associated with lower comfort in group learning settings, whereas higher self-efficacy was associated with greater comfort and, in turn, higher self-assessed learning success. These findings suggest that educational outcomes in group-based clinical skills training are not only determined by pedagogical design but are also influenced by learners’ psychological dispositions and their perceived capability to master specific clinical tasks.

A central finding of this study is the relevance of self-efficacy in a practical, socially embedded learning environment. Self-efficacy may be particularly important in physical examination training because students are required to integrate theoretical knowledge, manual skills, communication, and professional behavior while being observed by peers, tutors, and standardized patients. In such settings, students’ belief in their own ability to perform the required examination skills may influence how actively they participate, how they respond to feedback, and how willing they are to engage in repeated practice. Thus, self-efficacy may serve as a key mechanism linking students’ psychological characteristics to their subjective learning experience.

Our study confirmed the negative relationship between neuroticism and self-efficacy previously reported. Baranczuk (2021) delivers several potential explanations for this association which are of relevance in the setting of the present study. Persons with high neuroticism may perceive a situation with performance pressure and the possibility of making mistakes in front of others as difficult, stressful, and overwhelming; they may perceive themselves as unable to cope with this challenge and thus develop a lower self-efficacy. Conceivably, this leads to active avoidance of further comparable situations and therefore maintains the insufficient development of coping skills. Although the relationship in our study is correlational in nature, we assume that a lower self-efficacy may thus be a consequence of higher neuroticism and, in addition to the personality trait, may foster individual discomfort in group learning settings.

By drawing attention to the interplay between personality traits, self-efficacy, and perceived learning success, our study contributes to the growing discussion on psychological safety and learner-centered design in medical education. Small-group learning formats are widely used because they promote active participation, peer interaction, feedback, and experiential learning. However, our findings indicate that these formats may not be equally beneficial for all students. Learners with higher neuroticism or lower self-efficacy may experience group-based clinical skills training as less comfortable, which may limit their engagement and reduce their perceived learning success.

These findings are particularly relevant for the teaching of physical examination skills. Physical examination is not only a cognitive or technical task; it also requires students to perform in close interpersonal contact with patients or standardized patients, often under direct observation. Therefore, discomfort in group settings may have practical consequences for skill acquisition. Students who feel insecure or exposed may participate less actively, avoid volunteering, or benefit less from feedback opportunities. In contrast, students with higher self-efficacy may be more likely to engage in deliberate practice and to interpret feedback as helpful rather than threatening.

To ensure equity and effectiveness in clinical skills training, curriculum development should intentionally address these differences between learners. This could include integrating elements that foster psychological safety, such as structured debriefings, explicit norms for respectful peer interaction, transparent expectations, and instructor modeling of constructive feedback and vulnerability [33]. A “Safe Container” for learning in simulation offers a useful framework for creating learning spaces in which students feel secure enough to take interpersonal and academic risks [34]. Such approaches may be especially valuable in physical examination courses, where students must combine technical performance with communication and professional behavior in front of others.

Importantly, interventions designed to support students with lower self-efficacy or higher discomfort in group learning settings should not be understood as remedial measures for a small subgroup of learners. Rather, strategies such as scaffolded peer interaction, formative feedback, opportunities for repeated low-stakes practice, and structured reflection may benefit all students. These approaches align with a broader shift in medical education towards adaptive and inclusive learning environments that account for individual variability in students’ psychological and social contexts [35].

The findings reinforce the need to view medical students not only as learners acquiring technical competence, but also as individuals navigating complex emotional and interpersonal demands. Addressing these dimensions through curricular and instructional strategies may enhance both learning outcomes and well-being. Future research should further examine how personality traits and self-efficacy interact over time and whether targeted educational interventions can strengthen self-efficacy, reduce discomfort, and support more equitable participation in group-based clinical skills training. In particular, longitudinal studies could clarify whether increased self-efficacy in physical examination leads to greater comfort and improved objective performance, or whether positive learning experiences themselves contribute to the development of self-efficacy.

### Limitations

Several limitations should be considered when interpreting the findings of this study. First, the study relied on subjective measures of comfort and self-assessed learning success. Although these outcomes are highly relevant for understanding students’ experiences in group-based clinical skills training, they do not allow conclusions about objective competence or actual performance gains. Self-assessed learning success is clearly a psychological variable; it is the subjective feeling of how much competence one has gained during the course. The present study thus cannot answer the question whether students with a higher self-assessed learning success performed better than others. Instead, we suggest that self-assessed learning success and self-efficacy may contribute to the “feeling of preparedness”, which has been described as being important for job beginners, especially with respect to performance and persistence [36]. Thus, self-assessed learning success is probably less indicative of the actual ability of a student, but more of his or her potential to take up the job.

Second, data collection took place in the context of the COVID-19 pandemic, which represents an important contextual limitation. The participating cohort began medical school during a period characterized by online teaching, reduced in-person interaction, and limited opportunities for hands-on clinical practice. These circumstances may have influenced both students’ self-efficacy and their comfort in group-based learning settings. For example, prolonged periods of remote learning and social isolation may have reduced students’ confidence in practical clinical skills or increased discomfort in socially demanding learning environments. Therefore, the findings may partly reflect pandemic-related educational and social experiences rather than stable characteristics of medical students more generally.

Third, our cohort may not be representative of all medical students or of cohorts trained under regular curricular conditions. The specific timing of medical training during the pandemic limits the generalizability of the results. Follow-up studies with more recent cohorts who experienced less disruption in their medical education are therefore needed to determine whether the observed associations between personality traits, self-efficacy, comfort, and perceived learning success remain stable in post-pandemic learning environments. If such follow-up data are available, this should be explicitly reported; if not, this represents an important direction for future research.

Finally, the practical implications derived from this study should be interpreted with caution. While our findings suggest that clinical skills training may benefit from learner-sensitive adaptations—such as scaffolded practice, structured feedback, and measures to enhance psychological safety - the present study did not evaluate the effectiveness, feasibility, or resource requirements of such curricular modifications. Implementing these approaches may require additional staff time, tutor training, or organizational resources. At the same time, more targeted support and more efficient learning structures may ultimately improve the use of educational resources. Future intervention studies should therefore examine not only educational outcomes, but also the feasibility, acceptability, and resource implications of implementing such adaptations in clinical skills curricula.

## Conclusion

This study examined how personality traits and self-efficacy are associated with students’ comfort and self-assessed learning success in group-based clinical skills training. The findings suggest that students with higher neuroticism or lower self-efficacy may experience observed peer-based learning situations as more challenging, whereas higher self-efficacy appears to be associated with greater comfort and more positive perceived learning outcomes.

These results have practical implications for the design of physical examination training. Educators can foster psychologically safe learning environments by establishing clear norms for respectful peer interaction, normalizing mistakes as part of learning, providing structured and behavior-focused feedback, and offering repeated low-stakes opportunities for practice. Students who struggle with discomfort or low self-efficacy may particularly benefit from scaffolded learning sequences, beginning with observation and guided practice before progressing to independent demonstration in front of others.

Supporting self-efficacy through positive reinforcement, peer modeling, constructive feedback, and early mastery experiences may help students engage more actively in clinical skills training. Such strategies are likely to benefit not only students with higher neuroticism or lower self-efficacy, but all learners. However, as this study assessed perceived learning success rather than objective clinical performance, future research should include measures such as OSCE scores or standardized examiner ratings to examine whether these associations also translate into measurable gains in physical examination competence.

## Supplementary Information

Supplementary Material 1.

## Data Availability

The datasets used and analysed during the current study are available from the corresponding author on reasonable request.
