# Supplementary material for: Psychological determinants of successful practical teaching: personality traits, self-efficacy, and subjective perception in a hands-on clinical skills course
Source: BMC Med Educ. 2026 Jul 2;26:1069. doi: 10.1186/s12909-026-09788-2 (PMC13330428; doi:10.1186/s12909-026-09788-2)
Supplement: Supplementary file 1 — Supplementary Material 1. [file 12909_2026_9788_MOESM1_ESM.docx]

**Appendix 1: Structure and exemplary learning objectives of the course:**

**Appendix 2: NEO-FFI-30**

| **Item No.** | **Scale** | **Deutsch** | **English** |
| --- | --- | --- | --- |
| 1 | E | Ich habe gern viele Leute um mich herum. | I like to have a lot of people around me. |
| 2 | C | Wenn ich eine Verpflichtung eingehe, so kann man sich auf mich bestimmt verlassen. | When I make a commitment, I can be counted on to follow through. |
| 3 | C | Ich halte meine Sachen ordentlich und sauber. | I keep my belongings neat and clean. |
| 4 | N | Ich fühle mich anderen oft unterlegen | I often feel inferior to others. |
| 5 | E | Ich bin leicht zum Lachen zu bringen. | I laugh easily. |
| 6 | O | Ich finde philosophische Diskussionen langweilig. | I find philosophical discussions boring. |
| 7 | A | Ich bekomme häufiger Streit mit meiner Familie und meinen Kollegen. | I often get into arguments with family members and coworkers. |
| 8 | C | Ich kann mir meine Zeit recht gut einteilen, sodass ich meine Angelegenheiten rechtzeitig beende. | I am good at managing my time so that I finish things on time. |
| 9 | N | Wenn ich unter starkem Stress stehe, fühle ich mich manchmal, als ob ich zusammenbräche. | When I am under a great deal of stress, sometimes I feel like I’m going to pieces. |
| 10 | O | Mich begeistern die Motive, die ich in der Kunst und in der Natur finde. | I am intrigued by the patterns I find in art and nature. |
| 11 | A | Manche Leute halten mich für selbstsüchtig und selbstgefällig. | Some people think I am selfish and egotistical. |
| 12 | C | Ich versuche, alle mir übertragenen Aufgaben sehr gewissenhaft zu erledigen. | I strive to complete all my tasks conscientiously. |
| 13 | N | Ich fühle mich oft angespannt und nervös. | I often feel tense and jittery. |
| 14 | E | Ich bin gerne im Zentrum des Geschehens. | I like to be where the action is. |
| 15 | O | Poesie beeindruckt mich wenig oder gar nicht. | Poetry has little or no effect on me. |
| 16 | A | Im Hinblick auf die Absichten anderer bin ich eher zynisch und skeptisch. | I am skeptical and cynical about the motives of others. |
| 17 | N | Manchmal fühle ich mich völlig wertlos. | At times I feel utterly worthless. |
| 18 | E | Ich habe oft das Gefühl, vor Energie überzuschäumen. | I often feel bursting with energy. |
| 19 | E | Ich bin ein fröhlicher, gutgelaunter Mensch. | I am a cheerful, high-spirited person. |
| 20 | A | Manche Leute halten mich für kalt und berechnend. | Some people think I am cold and calculating. |
| 21 | N | Zu häufig bin ich entmutigt und will aufgeben, wenn etwas schiefgeht. | Too often, when things go wrong, I get discouraged and feel like giving up. |
| 22 | O | Wenn ich Literatur lese oder ein Kunstwerk betrachte, empfinde ich manchmal ein Frösteln oder eine Welle der Begeisterung. | Sometimes when I read poetry or look at a work of art, I feel a chill or wave of excitement. |
| 23 | O | Ich habe wenig Interesse, über die Natur des Universums oder die Lage der Menschheit zu spekulieren. | I have little interest in speculating on the nature of the universe or the human condition. |
| 24 | A | Ich versuche stets rücksichtsvoll und sensibel zu handeln. | I try to be courteous and considerate to everyone. |
| 25 | C | Ich bin eine tüchtige Person, die ihre Arbeit immer erledigt. | I am a productive person who always gets the job done. |
| 26 | N | Ich fühle mich oft hilflos und wünsche mir eine Person, die meine Probleme löst. | I often feel helpless and want someone else to solve my problems. |
| 27 | E | Ich bin ein sehr aktiver Mensch. | I am a very active person. |
| 28 | C | Ich werde wohl niemals fähig sein, Ordnung in mein Leben zu bringen. | I’ll probably never be able to get my life together. |
| 29 | O | Ich habe oft Spaß daran, mit Theorien oder abstrakten Ideen zu spielen. | I enjoy playing with theories or abstract ideas. |
| 30 | A | Um zu bekommen, was ich will, bin ich notfalls bereit, Menschen zu manipulieren. | To get what I want, I am sometimes willing to manipulate others. |

N= Neuroticism, E = Extraversion, O = Openness, A = Agreeableness,

C = Conscientiousness.

**Appendix 3: General Self-Efficacy Scale**

| Item No. | Deutsch | English |
| --- | --- | --- |
| 1 | Wenn sich Widerstände auftun, finde ich Mittel und Wege, mich durchzusetzen. | When I face resistance, I find ways to assert myself. |
| 2 | Die Lösung schwieriger Probleme gelingt mir immer, wenn ich mich darum bemühe. | I can always solve difficult problems if I try hard enough. |
| 3 | Es bereitet mir keine Schwierigkeiten, meine Absichten und Ziele zu verwirklichen. | I have no trouble achieving my goals and intentions. |
| 4 | In unerwarteten Situationen weiß ich immer, wie ich mich verhalten soll. | In unexpected situations, I always know how to act. |
| 5 | Auch bei überraschenden Ereignissen glaube ich, dass ich gut mit ihnen zurechtkommen kann. | Even in surprising events, I believe I can cope well. |
| 6 | Schwierigkeiten sehe ich gelassen entgegen, weil ich meinen Fähigkeiten immer vertrauen kann. | I face difficulties calmly because I always trust my abilities. |
| 7 | Was auch immer passiert, ich werde schon klarkommen. | No matter what happens, I will manage somehow. |
| 8 | Für jedes Problem kann ich eine Lösung finden. | I can find a solution to any problem. |
| 9 | Wenn eine neue Sache auf mich zukommt, weiß ich, wie ich damit umgehen kann. | When something new comes up, I know how to handle it. |
| 10 | Wenn ein Problem auftaucht, kann ich es aus eigener Kraft meistern. | When a problem arises, I can overcome it on my own. |

**Appendix 4: Comfort in group learning settings**

| Item No. | Deutsch | English |
| --- | --- | --- |
| 1 | Ich fühle mich wohl bei der Durchführung praktischer Übungen vor einer mir teils unbekannten Gruppe. | I feel comfortable performing practical exercises in front of a group that is partly unknown to me. |
| 2 | Ich fühle mich wohl bei der Durchführung von Untersuchungen an meinen Kommilitonen. | I feel comfortable performing examinations on my fellow students. |
| 3 | Ich fühle mich wohl bei der Durchführung von Untersuchungen an Simulationspersonen. | I feel comfortable performing examinations on simulated patients. |
| 4 | Ich fühle mich wohl dabei, Feedback von einer teils unbekannten Gruppe entgegenzunehmen. | I feel comfortable receiving feedback from a group that is partly unknown to me. |

**Appendix 5: Learning Success**

| **Item No.** | **Deutsch** | **English** |
| --- | --- | --- |
|  | Wie sicher fühlen Sie sich... | How confident do you feel... |
| 1 | ...bei der körperlichen Untersuchung eines Patienten? | ...when performing a physical examination of a patient? |
| 2 | ...bei der Durchführung eines Anamnesegesprächs? | ...when conducting a medical history interview? |
| 3 | ...bei der Untersuchung der Lymphknoten? | ...when examining lymph nodes? |
| 4 | ...bei der Untersuchung der Schilddrüse? | ...when examining the thyroid gland? |
| 5 | ...bei der Durchführung einer orthopädischen Basisuntersuchung? | ...when performing a basic orthopedic examination? |
| 6 | ...bei der Durchführung einer neurologischen Basisuntersuchung? | ...when performing a basic neurological examination? |
| 7 | ...bei der Untersuchung des Herzens und der Lunge? | ...when examining the heart and lungs? |
| 8 | ...bei der Untersuchung des Abdomens? | … when examining the abdomen? |
